# Higher Peripheral Cholesterol and a Positive Correlation With Risk for Large-For-Gestational-Age Neonates in Pre-Pregnancy Underweight Women

**DOI:** 10.3389/fendo.2021.760934

**Published:** 2021-11-24

**Authors:** Dongyu Wang, Wenjing Ding, Chengcheng Ding, Haitian Chen, Weihua Zhao, Bo Sun, Zilian Wang

**Affiliations:** ^1^ Department of Obstetrics and Gynecology, The First Affiliated Hospital of Sun Yat-sen University, Guangzhou, China; ^2^ Department of Obstetrics and Gynecology, Shenzhen Second People’s Hospital, Shenzhen, China

**Keywords:** underweight, cholesterol, LGA large for gestational age, SGA (small for gestational age), overweight

## Abstract

**Objective:**

As the high proportion of underweight pregnant women, omission of their weight gain and blood lipids management during gestation might lead to adverse pregnancy outcomes. This study aimed to determine the relationship between lipid profile and risks for adverse pregnancy outcomes in pre-pregnancy underweight women.

**Methods:**

This study was part of an ongoing cohort study including Chinese gravidas delivered from January 2015 to December 2016. Included subjects were grouped into underweight, normal-weight, and overweight by BMI before conception. Logistic regression was used to assess the association between lipid profiles during second trimester and adverse obstetric outcomes in each group. A subgroup analysis according to the gestational weight gain, in which subjects in each group were divided into above and within the Institute of Medicine (IOM) recommendations, was performed.

**Results:**

A total of 6, 223 women were included. The proportion of underweight (19.3%) was similar to that of overweight women (19.4%) in South China. Peripheral total cholesterol (TC) level in underweight women was significantly higher than that in overweight women (P <0.001). After adjusting maternal age, TC level was positively correlated to the risk for large-for-gestational-age (LGA) [aOR =2.24, 95%CI (1.08, 4.63)], and negatively related to the risk for small-for-gestational age (SGA) [aOR =0.71, 95%CI (0.59, 0.85)] in underweight women, but not in normal-weight or overweight women. The subgroup analysis showed that maternal TC level was positively correlated with the risk of LGA only in underweight women who gained weight more than the IOM recommendations.

**Conclusion:**

Underweight pregnant women with high TC levels had a higher risk for LGA, especially among women whose gestational weight gain were above the IOM recommendations. Therefore, clinical management of lipids and weight gain during gestation should also be recommended for underweight women.

## Introduction

Maternal overweight and obesity have drawn worldwide attention increasingly in recent years. The 2011-2012 US National Health and Nutrition Examination Survey (NHANES) data showed that the prevalence of obesity in women aged 20-39 years was 31.8% in the United State States (1). Furthermore, 48% and 24% of women were overweight before conception in the US and Germany, respectively, among singleton term nulliparous women. In UK, about one-quarter of women aged over 20 years were diagnosed with obesity, according to the European Peristat Database and WHO data. However, in Asia, the proportion of obesity among women of childbearing age is not as high as in Western countries. In South Korea, the proportion of women of reproductive age whose body mass index (BMI) were more than 30 kg/m² was less than 10%. A similar condition is found in China, especially in south China. Using the cutoff for inclusion in overweight/obesity in Asia-Pacific countries (overweight: BMI ≥23 kg/m2; obesity: BMI ≥25 kg/m2), 15.8% of pregnant women were diagnosed with obesity at the first antenatal visit in Hong Kong. Although 10-24% of pregnant women were reported to be overweight/obesity, a similar proportion (11-13%) of underweight pregnant women were also reported in China (2).

A high BMI is associated with increased risks of developing pregnancy comorbidities such as gestational diabetes mellitus (GDM), preeclampsia and cesarean section. Considering the lower prevalence of obesity in China than that in western countries, the incidences of pregnancy complications were assumed to be lower in Chinese pregnant women. However, the facts are on the contrary. The occurrence of GDM in China is higher than that in many western countries, such as Northern California (3), Louisiana (4) and Canada (5). Thus, the risk factors for GDM or other complications in underweight women might be different from those in women with obesity.

Dyslipidemia is a well-known risk factor of adverse pregnancy outcomes, including GDM and large-for-gestational gestational age (LGA) (6). Maternal triglyceride (TG) levels were elevated throughout pregnancy, whereas high-density lipoprotein cholesterol (HDL-c) levels were reduced during the late trimester, in women with GDM compared with women without GDM (7). A large community-based cohort study demonstrated that serum TG levels during the first trimester of pregnancy were positively associated with adverse obstetric outcomes, including gestational hypertension, preeclampsia and LGA (8). Another retrospective study in China showed that total cholesterol (TC) level during early pregnancy was an independent risk factor for GDM, TG level for GDM and preeclampsia, and low-density lipoprotein cholesterol (LDL-c) level for GDM and preterm birth (9). However, rare studies were concerned about whether the associations between maternal and fetal morbidities and lipid profiles vary in different pre-gestational BMI categories.

Therefore, a retrospective cohort study was performed to evaluate the correlations between maternal lipid profiles and adverse pregnancy outcomes after grouping pregnant women by pre-pregnancy BMI as underweight, normal-weight and overweight women.

## Method

### Study Population

This study was part of an ongoing cohort study in which subjects were recruited at the first prenatal visit and followed standard obstetric management until 3 days after delivery in the First Affiliated Hospital of Sun Yat-sen University. We retrospectively included Chinese pregnant women, delivered from January 2015 to December 2016, at the time of antepartum screening for GDM. Inclusion criteria were: 1) singleton pregnancy without known chronic non-communicable diseases (except obesity); 2) attending regular prenatal care in our hospital, and 3) had integrated medical records. Women were excluded if they had any of the following conditions: multiple pregnancy, preexisting hypertension, preexisting diabetes, diseases of immune system or other diseases that may influence the lipid profile, taking drugs that affected lipid levels, i.e. glucocorticoids, before or during pregnancy and missing data on crucial items like height, pre-pregnancy and pre-partum weight, lipids profile during second trimester, 75g oral glucose tolerance test (OGTT) results and pregnancy outcomes.

### Data Collection

A file including demographic and medical records was collected. Demographic data included maternal age, gravidity, parity, height, pre-pregnancy weight and gestational weight gain (GWG). Besides, medical information, including lipid profiles (TC, TG, HDL-c, and LDL-c) during the second trimester of pregnancy, OGTT results, pregnancy complications (GDM and preeclampsia), and obstetric outcomes (postpartum hemorrhage, LGA, small-for-gestational-age e (SGA), and preterm birth), were also collected.

### Biochemical Parameters Measurement

Peripheral blood samples for lipid profiles measurement were obtained between 08:00 and 09:00 in the morning after overnight fasting. Biochemical parameters including TC, TG, HDL-c, and LDL-c were measured with standard enzymatic procedures on an automatic chemistry analyzer (Abbott Aeroset, Chicago, IL, USA).

### Definition

Pre-pregnancy body mass index (pre-BMI) was calculated as dividing the pre-pregnancy body weight (kilograms) by the square of height (meters). Included subjects were classified into underweight (<18.5 kg/m2), normal-weight (18.5-22.9 kg/m2) and overweight (≥23 kg/m2) based on the Asia-Pacific classification of BMI (10). The GWG guidelines published by the Institute of Medicine (IOM) recommends a GWG of 12.70 – 18.14 kg (28 – 40 lb) for underweight women, 11.34 – 15.88 kg (25 – 35 lb) for normal-weight women, and 6.80 – 11.34 kg (15 – 25 lb) for overweight women (11). Women in each group were divided into two subgroups according to the total GWG which was categorized as above and within the IOM recommendations.

According to the diagnostic criteria, which was founded by the Chinese Medical Association in 2014 (12), GDM was diagnosed when any serum glucose value equaled or exceeded the thresholds during OGTT:fasting blood glucose (FBG), 5.1 mmol/l; 1 hour, 10.0mmol/l; and 2 hour, 8.5mmol/l. However, FBG ≥7.0mmol/l or 2-hour result ≥ 11.1mmo/l was considered as pre-gestational diabetes mellitus and then excluded from our study.

Preeclampsia was defined as new-onset hypertension (systolic blood pressure ≥140 mmHg and/or diastolic blood pressure ≥90 mmHg) with albuminuria (≥0.3g protein of urine in 24 hours or positive detection of random urine protein) or other biochemical or hematological abnormalities (13).

Postpartum hemorrhage was defined by the International Federation of Gynecology and Obstetrics (14) criteria (24 hours postpartum bleeding ≥500 ml in vaginal delivery or ≥1000 ml in cesarean delivery).

Small for gestational age (SGA) was defined as neonatal birth weight less than the 10th percentile for gestational age, and LGA was above the 90th percentile (15).

Preterm birth was gestational age at delivery smaller than 37 weeks (16).

### Statistical Analysis

Data analyses were performed by the SPSS 20.0 (Inc., Chicago, IL, USA). Normal continuous variables were presented as means ± standard deviation (SD), non-normal continuous variables were presented as median (interquartile), and categorical variables were presented as number (percentage). Differences between groups were assessed by Mann-Whitney U test, Kruskal Wallis test or Chi-square test as appropriate. Logistic regression was used to assess the association between lipid profiles during second trimester and risks for pregnancy complications or adverse obstetric outcomes. Crude and adjusted analysis was performed using maternal age as a continuous variable. P value less than 0.05 was considered statistically significant. 

## Results

### Basic Characteristics and Obstetric Outcomes

**Figure 1:**
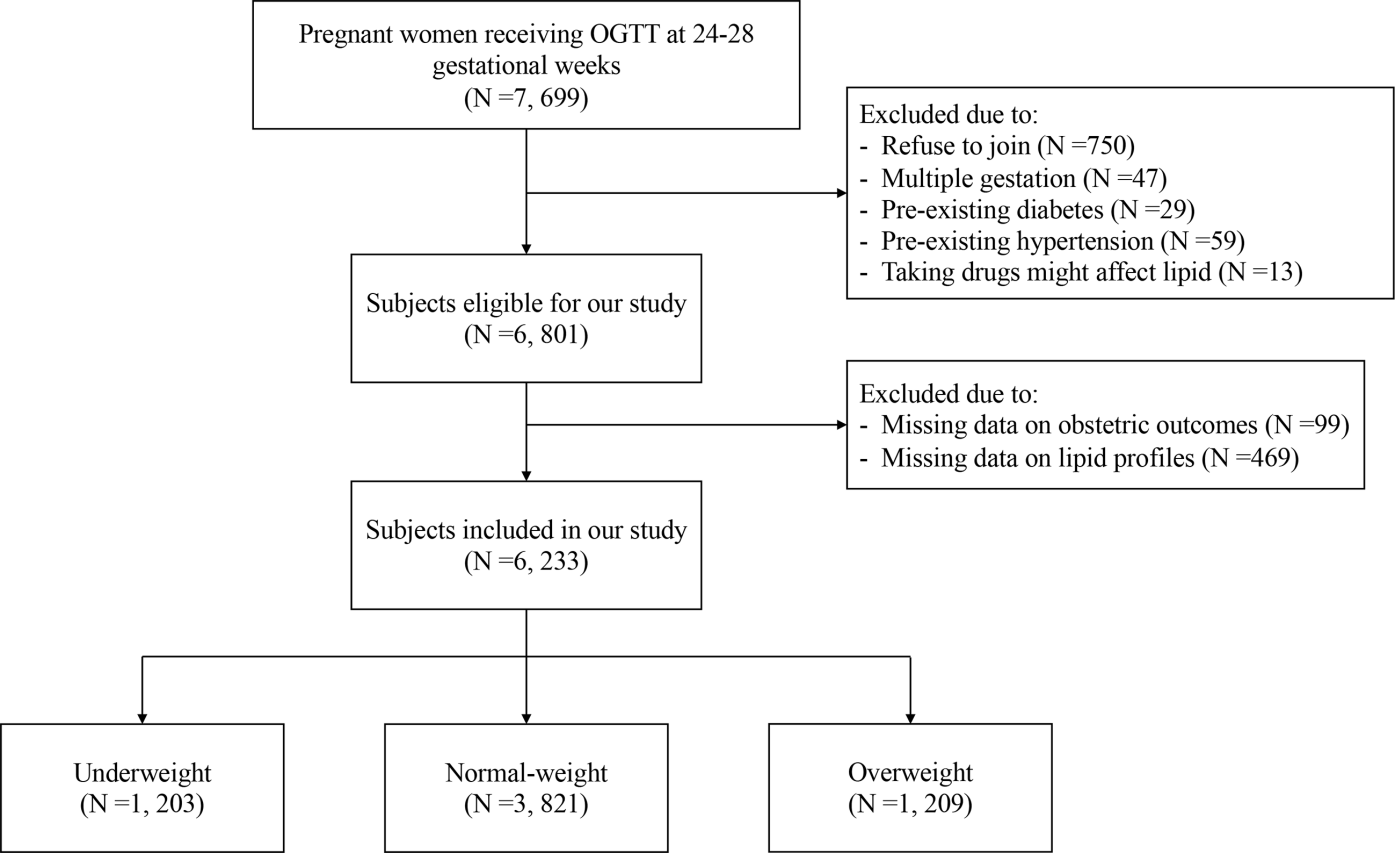
Flow chart of selecting eligible subjects. OGTT, oral glucose tolerance test.

A total of 7, 699 pregnant women delivered in our hospital during January 2015 and December 2015, and 6, 233 women were included in this study. The detailed screening procedure is shown in **Figure 1**. The proportion of underweight, normal-weight and overweight women was 19.3% (1, 203/6, 233), 61.3% (3, 821/6, 233) and 19.4% (1, 209/6, 233), respectively. **Table 1** showed the basic characteristics of included subjects. There were significant differences in maternal age among three groups (P <0.001), thus the potential effects of maternal age were adjusted in the following analysis. Gestational weight gain of overweight women was significantly lower than that of underweight and normal-weight women (P <0.001). Significantly increased incidences of GDM and LGA were found in women with higher pre-pregnancy BMI (P <0.001), whereas a significantly decreased incidence of SGA was found in women with higher pre-pregnancy BMI (P <0.001). The incidence of preeclampsia in overweight women was significantly higher than that in underweight and normal-weight women (P <0.001), and the incidence of postpartum hemorrhage in underweight women was significantly lower than that in normal-weight women (P =0.007). No significant differences were found in other comparisons.

**Table 1:** Basic characteristics and obstetric outcomes of subjects included in the study (N =6, 233).

	Underweight	Normal-weight	Overweight	P value
N (% of total subjects)	1, 203 (19.3%)	3, 281 (61.3%)	1, 209 (19.4%)	
Maternal age	29.5 ± 3.9	31.4 ± 4.3	32.6 ± 4.2	<0.001^abc^
Gravity	2.2 ± 1.2	2.1 ± 1.2	2.1 ± 1.2	0.532
Parity	0.5 ± 0.6	0.5 ± 0.6	0.4 ± 0.6	0.608
Height	1.60 ± 0.05	1.60 ± 0.05	1.60 ± 0.05	0.428
Pre-pregnancy weight	45.1 ± 3.2	52.8 ± 4.5	64.2 ± 7.1	<0.001^abc^
Pre-pregnancy BMI	17.5 ± 0.8	20.6 ± 1.2	25.1 ± 2.2	<0.001^abc^
GWG	13.7 ± 4.6	13.3 ± 4.3	11.4 ± 4.7	<0.001^bc^
GDM (% of each group)	205 (17.0%)	689 (21.0%)	355 (29.4%)	<0.001^abc^
Preeclampsia (% of each group)	30 (2.5%)	105 (3.2%)	104 (8.6%)	<0.001^bc^
Preterm birth (% of each group)	83 (6.9%)	266 (8.1%)	114 (9.4%)	0.219
PPH (% of each group)	54 (4.5%)	256 (7.8%)	80 (6.6%)	0.007^a^
LGA (% of each group)	23 (1.9%)	177 (5.4%)	89 (7.4%)	<0.001^abc^
SGA (% of each group)	200 (16.6%)	318 (9.7%)	70 (5.8%)	<0.001^abc^

BMI, body mass index; GWG, gestational weight gain; GDM, gestational diabetes mellitus; PPH, postpartum hemorrhage; LGA, large for gestational age; SGA, small for gestational age.

^a, b, c^indicates a significant difference between underweight and normal weight, underweight and overweight, and normal weight and overweight, respectively.

**Table 2** showed the biochemical biomarkers, including lipid profiles and OGTT results during second trimester, in underweight, normal-weight and overweight women respectively. As our previous study suggested that TG/HDL-c ratio could be a good marker to predict the risks of GDM and LGA (17), TG/HDL-c ratio was also included in our analysis. Concentration of TC of overweight women was significantly lower than that of underweight and normal-weight women (P <0.001). Concentrations of TG, fasting blood glucose, 1-hour and 2-hour blood glucose of OGTT in pre-pregnancy underweight women were significantly lower than those in normal-weight and overweight women (P <0.001), whereas HDL-c level in underweight women was significantly higher than that in normal-weight and overweight women (P <0.001). Concentration of LDL-c in overweight women was significantly lower than that in normal-weight women (P =0.024).

**Table 2:** Maternal biochemical markers during second trimester stratified by pre-pregnancy BMI.

	Underweight	Normal-weight	Overweight	P value
TC	6.55 ± 1.16	6.49 ± 1.10	6.29 ± 1.18	<0.001^bc^
TG	2.10 ± 1.61	2.30 ± 0.91	2.61 ± 1.30	<0.001^abc^
HDL-c	2.10 ± 0.37	2.03 ± 0.36	1.94 ± 0.35	<0.001^abc^
LDL-c	3.69 ± 0.80	3.68 ± 1.61	3.53 ± 0.74	0.024^c^
TG/HDL-c	1.03 ± 0.53	1.19 ± 0.67	1.42 ± 0.81	<0.001^abc^
OGTT-0h	4.32 ± 0.35	4.42 ± 0.37	4.57 ± 0.45	<0.001^abc^
OGTT-1h	7.51 ± 1.66	7.87 ± 1.64	8.33 ± 1.66	<0.001^abc^
OGTT-2h	6.76 ± 1.42	6.99 ± 1.37	7.28 ± 1.35	<0.001^abc^

BMI, body mass index; TC, total cholesterol; TG, triglycerides; HDL-c, high-density lipoprotein cholesterol; LDL-c, low-density lipoprotein cholesterol; OGTT, oral glucose tolerance test.

^a, b, c^indicates a significant difference between underweight and normal weight, underweight and overweight, and normal weight and overweight, respectively.

### Correlations Between Maternal Lipids and Obstetric Outcomes Stratified by Pre-Pregnancy BMI

**Figure 2:**
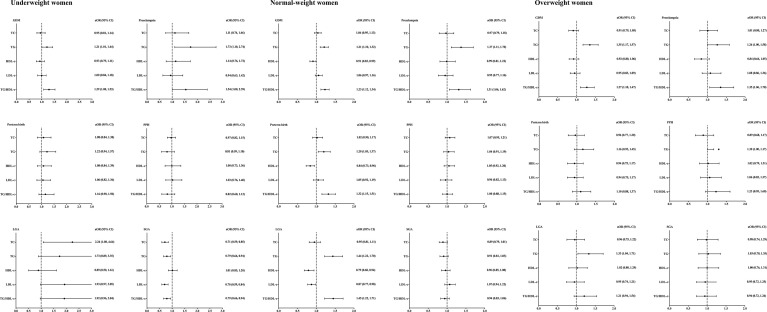
Lipid profiles during second trimester and pregnancy complications stratified by pre-pregnancy BMI. BMI, body mass index; aORs, adjusting odd ratios; CI, confidence interval; GDM, gestational diabetes; PPH, postpartum hemorrhage; LGA, large for gestational age; SGA,small for gestational age. Included subjects were grouped into underweight (<18.5 kg/m^2^), normal-weight (18.5-22.9 kg/m^2^), and overweight (≥23 kg/m^2^) by BMI before conception based on the WHO criteria for Asian populations. All ORs were adjusted for maternal age.

Correlations between maternal lipid profiles during second trimester and adverse pregnancy outcomes were displayed in **Figure 2**. All odds ratios were adjusted for maternal age.

Among underweight women, TG/HDL-c ratio was found to be positively correlated with the incidence of GDM [aOR =1.29, 95% CI (1.08, 1.53)]. TG level [aOR =1.73, 95% CI (1.10, 2.74)] and TG/HDL-c ratio [aOR =1.54, 95% CI (1.00, 2.39)] were positively correlated with the incidence of preeclampsia. TC level was positively correlated with LGA incidence [aOR =2.24, 95% CI (1.08, 4.64)]. The incidence of SGA was found to be negatively correlated with TC [aOR =0.71, 95% CI (0.59, 0.85)], TG [aOR =0.79, 95% CI (0.66, 0.94)], LDL-c [aOR =0.70, 95% CI (0.59, 0.84)] and TG/HDL-c ratio [aOR =0.79, 95% CI (0.66, 0.94)]. No significant correlations between lipid profiles and incidence of preterm birth/postpartum hemorrhage were found in underweight women.

Among normal-weight women, TG level was positively correlated with the incidence of GDM [aOR =1.21, 95% CI (1.10, 1.32)], preeclampsia [aOR =1.37, 95% CI (1.11, 1.70)], preterm birth [aOR =1.20, 95% CI (1.05, 1.37)] and LGA [aOR =1.44, 95% CI (1.22, 1.70)]. The ratio of TG/HDL-c was positively correlated with the incidence of GDM [aOR =1.23, 95% CI (1.12, 1.34)], preeclampsia [aOR =1.31, 95% CI (1.06, 1.62)], preterm birth [aOR =1.32, 95% CI (1.15, 1.51)] and LGA [aOR =1.45, 95% CI (1.22, 1.71)]. There were no significant associations between TC, HDL-c or LDL-c and pregnancy complications in normal-weight women.

Among overweight women, TG level was found to be positively correlated with the incidence of GDM [aOR =1.35, 95% CI (1.17, 1.57)], preeclampsia [aOR =1.26, 95% CI (1.00, 1.58)] and LGA [aOR =1.33, 95% CI (1.04, 1.71)]. Positive correlations between TG/HDL-c ratio and incidence of GDM [aOR =1.27, 95% CI (1.10, 1.47)] and preeclampsia [aOR =1.35, 95% CI (1.06, 1.70)] were found in overweight women.

### Subgroup Analysis According to Gestational Weight Gain

Since the GWG among underweight and normal-weight women were significantly higher than that among overweight women, we did a subgroup analysis according to GWG. Women in each group were divided into two subgroups according to the total GWG which was categorized as above and within the IOM recommendations.

Differences in biochemical parameters among each group were presented in **Table 3**. Among women with above GWG, no significant difference in TC level was found, while the concentrations of TG, fasting blood glucose, 1-hour and 2-hour blood glucose of overweight women were significantly higher than those of underweight and normal-weight women. Among women within GWG recommendations, maternal TC and HDL-c levels of underweight women were significantly higher than those of overweight women. Besides, concentrations of TG, fasting blood glucose, 1-hour and 2-hour blood glucose of underweight women were significantly lower than those of normal-weight and overweight women.

**Table 3:** Maternal biochemical markers during second trimester stratified by pre-pregnancy BMI.

	Underweight	Normal-weight	Overweight	P value
Above IOM recommended GWG				
N	136	834	606	
TC	6.58 ± 1.09	6.55 ± 1.05	6.38 ± 1.27	0.059
TG	2.12 ± 0.63	2.26 ± 0.78	2.54 ± 0.95	<0.001^bc^
HDL-c	2.05 ± 0.36	2.08 ± 0.37	1.97 ± 0.35	<0.001^c^
LDL-c	3.75 ± 0.81	3.67 ± 0.70	3.57 ± 0.76	0.035
TG/HDL-c	1.07 ± 0.40	1.14 ± 0.51	1.35 ± 0.63	<0.001^bc^
OGTT-0h	4.38 ± 0.48	4.42 ± 0.43	4.50 ± 0.43	0.006^c^
OGTT-1h	7.43 ± 1.58	7.66 ± 1.58	8.05 ± 1.63	<0.001^bc^
OGTT-2h	6.59 ± 1.32	6.71 ± 1.26	7.04 ± 1.26	<0.001^bc^
Within IOM recommended GWG				
N	1067	2447	603	
TC	6.55 ± 1.17	6.47 ± 1.12	6.20 ± 1.08	<0.001^bc^
TG	2.10 ± 1.70	2.31 ± 0.95	2.68 ± 1.58	<0.001^abc^
HDL-c	2.11 ± 0.38	2.02 ± 0.36	1.90 ± 0.33	<0.001^abc^
LDL-c	3.68 ± 0.80	3.68 ± 1.82	3.49 ± 0.72	0.089
TG/HDL-c	1.02 ± 0.54	1.21 ± 0.72	1.48 ± 0.96	<0.001^abc^
OGTT-0h	4.28 ± 0.47	4.42 ± 0.40	4.61 ± 0.56	<0.001^abc^
OGTT-1h	7.48 ± 1.76	7.93 ± 1.69	8.56 ± 1.75	<0.001^abc^
OGTT-2h	6.73 ± 1.54	7.06 ± 1.45	7.45 ± 1.49	<0.001^abc^

BMI, body mass index; TC, total cholesterol; TG, triglycerides; HDL-c, high-density lipoprotein cholesterol; LDL-c, low-density lipoprotein cholesterol; OGTT, oral glucose tolerance test.

^a, b, c^indicates a significant difference between underweight and normal weight, underweight and overweight, and normal weight and overweight, respectively.

**Figure 3:**
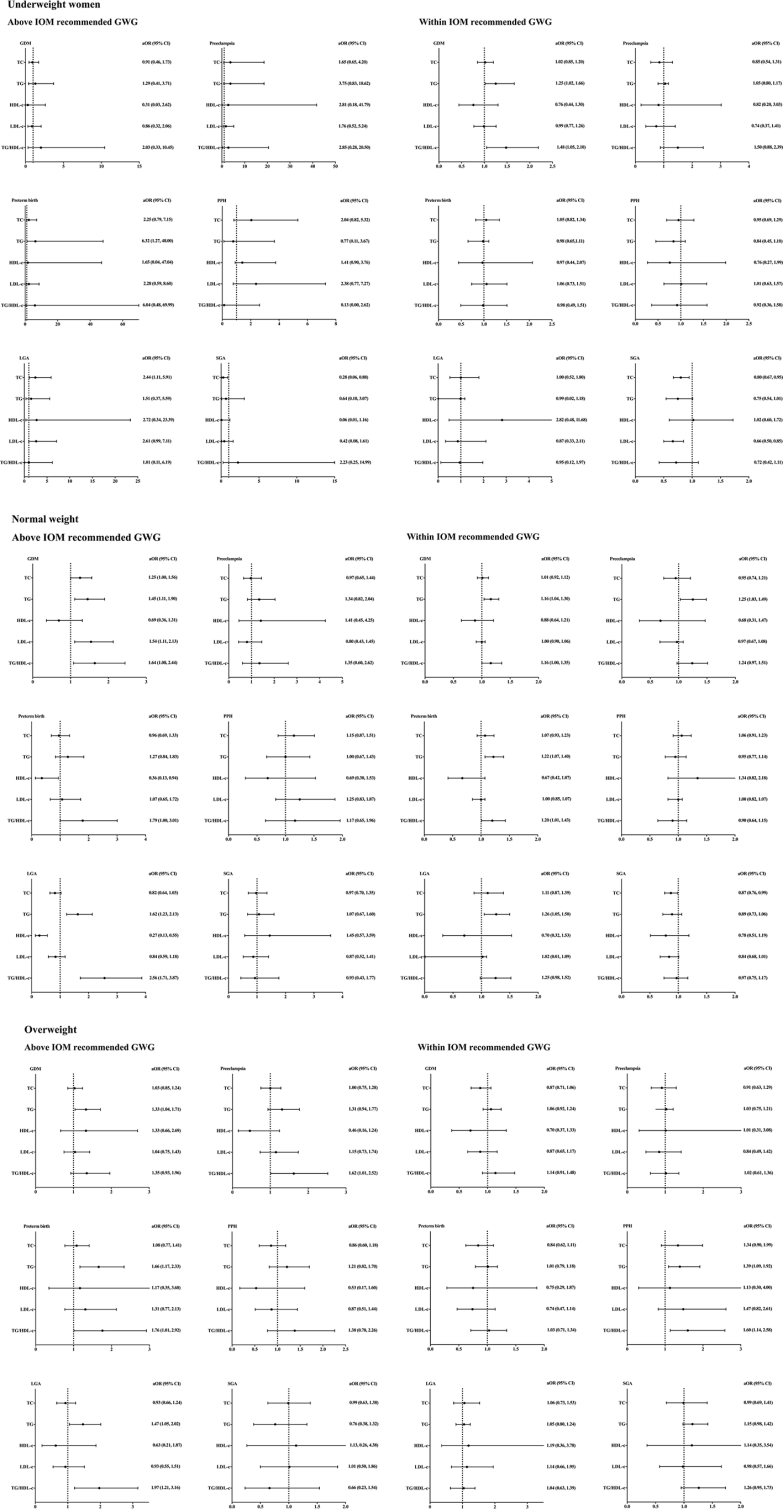
Lipid profiles during second trimester and pregnancy complications stratified by pre-pregnancy BMI and gestational weight gain. BMI, body mass index; aORs, adjusting odd ratios; IOM, Institute of Medicine; GWG, gestational weight gain; GDM, gestational diabetes mellitus; PPH, postpartum hemorrhage; LGA, large for gestational age; SGA, small for gestational age; TC, total cholesterol; TG, triglycerides; HDL-c, high-density lipoprotein cholesterol; LDL-c, low-density lipoprotein cholesterol. Included subjects were grouped into underweight (<18.5 kg/m^2^), normal-weight (18.5-22.9 kg/m^2^), and overweight (≥23 kg/m^2^) by BMI before conception based on the WHO criteria for Asian populations first. Subjects in each group were divided into two subgroups according to the total GWG which was categorized as above and within the IOM recommendations. All ORs were adjusted for maternal age.

**Figure 3** showed the correlations between lipid profiles and adverse pregnancy outcomes.

Among underweight women who gained more weight beyond the IOM recommendations, TG level was only found to be positively correlated with the incidence of preterm birth [aOR =6.32, 95% CI (1.27, 48.00)]. Meanwhile, TC was positively correlated with LGA incidence [aOR =2.44, 95% CI (1.11, 5.91)], but negatively correlated with SGA incidence [aOR =0.28, 95% CI (0.06, 0.88)]. The associations between maternal TC and the incidence of LGA and SGA remained after adjusting the GWG and maternal age (**Supplementary Tables 1** and **2**). Among underweight women within IOM recommended GWG, a positive correlation between GDM incidence and TG [aOR =1.25, 95% CI (1.02, 1.66)] and TG/HDL-c ratio [aOR =1.48, 95% CI (1.05, 2.10)] was found. Besides, TC level was found to be negatively correlated with SGA incidence [aOR =0.80, 95% CI (0.67, 0.95)], but no significant association with the incidence of LGA.

Among normal-weight women whose GWG were above the IOM recommendations, the concentrations of TC [aOR =1.25, 95% CI (1.00, 1.56)], TG [aOR =1.45, 95% CI (1.11, 1.90)], LDL-c [aOR =1.54, 95% CI (1.11, 2.13)] and TG/HDL-c ratio [aOR =1.64, 95% CI (1.08, 2.44)] were positively correlated with the incidence of GDM. HDL-c level was negatively correlated with the incidence of preterm birth [aOR =0.36, 95% CI (0.13, 0.94)]. TG level [aOR =1.62, 95% CI (1.23, 2.13)] and TG/HDL-c ratio [aOR =2.56, 95% CI (1.71, 3.87)] were positively correlated with the incidence of LGA, whereas HDL-c level was negatively correlated with LGA incidence [aOR =0.27, 95% CI (0.13, 0.55)]. No significant associations between lipids and incidence of preeclampsia, postpartum hemorrhage or SGA were observed. Among women within IOM recommended GWG, a positive correlation between GDM incidence and TG [aOR =1.16, 95% CI (1.04, 1.30)] and TG/HDL-c ratio [aOR =1.16, 95% CI (1.00, 1.35)] was found. In addition, TG level was found to be positively correlated with the incidence of preeclampsia [aOR =1.25, 95% CI (1.03, 1.49)], preterm birth [aOR =1.22, 95% CI (1.07, 1.40)] and LGA [aOR =1.26, 95% CI (1.05, 1.50)].

Among overweight women whose GWG were above the IOM recommendations, TG was found to be positively correlated with the incidence of GDM [aOR =1.33, 95% CI (1.04, 1.71)], preterm birth [aOR =1.66, 95% CI (1.17, 2.33)] and LGA [aOR =1.47, 95% CI (1.05, 2.02)]. The ratio of TG/HDL-c was positively correlated with the incidence of preeclampsia [aOR =1.62, 95% CI (1.01, 2.52), preterm birth [aOR =1.76, 95% CI (1.01, 2.92)] and LGA [aOR =1.97, 95% CI (1.21, 3.16)]. No significant association between other lipids and pregnancy complications was observed. On the other hand, there was no significant association between lipid profiles and pregnancy complications in overweight women within IOM recommended GWG.

## Discussion

### Main Findings

In the present study, we conducted a large retrospective cohort study to assess the association between lipid profiles during the second trimester and adverse pregnancy outcomes in women grouped by pre-pregnancy BMI. We found that, in South China, the proportion of pre-pregnancy underweight women (19.3%) was similar to that of overweight women (19.4%) according to the WHO criteria for Asian populations. Moreover, maternal TC levels in underweight women were significantly higher than normal-weight and overweight women. Maternal TC level was found to be positively correlated with the incidence of LGA and negatively correlated with the incidence of SGA in underweight but not in normal-weight or overweight women. Besides, a subgroup analysis according to GWG showed that the positive association between maternal TC concentration and LGA incidence was found in underweight women with higher GWG but not those within the IOM recommended GWG.

Our previous study showed that women in the BMI range of 25 to 28 kg/m^2^ are at high risks, similar to women with BMI ≧28kg/m^2^, for adverse obstetric outcomes, suggesting that a lower BMI cut-off for defining obesity would be better for pregnant women in China, especially in South China (18). Thus the WHO criteria specifically for Asian-Pacific countries were used in our study. The increasing prevalence of maternal obesity and related complications necessitates focusing clinical management on lipids profiles during gestation. The similar proportion of underweight to that of overweight women, but the still high incidence of pregnancy complications in our study, demonstrated that metabolic management might also be necessary for underweight women.

We found that circulating TC levels in underweight women were significantly higher than that in normal-weight and overweight women. Our finding was consistent with research in Pune, which showed that TC concentrations elevated during 18 and 28 gestational weeks in underweight women (19). Cholesterol acts as a precursor to many hormones, including estrogen and progesterone. Lassance et al. (20) found that serum concentrations of estradiol and progesterone were significantly lower in pregnant women with obesity (BMI 30–35 kg/m^2^) than in normal-weight pregnant women (BMI 19–25 kg/m^2^). Cholesterol is essential for fetal growth, steroid synthesis and neurodevelopment, supported by the observations that abnormal cholesterol metabolism was related to impaired neurodevelopment (21), low birthweight (22), and fetal growth restriction (23). The fetus is unable to synthesize cholesterol due to the immature liver and adrenal gland. Thus, the physiological hyperlipidemia could be an adaptive response to satisfy the increased fetal demand with advancing gestation (24, 25). Taken together with a significantly lower incidence of SGA in underweight than normal-weight and overweight women, we speculate that the increase in circulating TC levels might be a compensatory response to ensure the sufficient cholesterol supply to the fetal growth.

Our results demonstrated TC levels were positively correlated with the incidence of LGA and negatively correlated with the incidence of SGA in underweight but not in normal-weight or overweight women. Consistently, Kulkarni et al. reported that TC levels were positively related to the newborn birth size, including birth weight, abdominal circumference and mid-upper-arm circumference, in rural underweight women, and a 1-SD-higher TC during second trimester was associated with a 39-g-higher birthweight (19). A recent cohort study in Japan demonstrated that an increase in maternal TC was linearly associated with LGA and a decrease in maternal TC was associated with SGA after adjusting several confounders, including maternal age, pre-pregnancy BMI, GWG, and glucose level (26). However, inconsistent findings were reported. M.Mudd et al. demonstrated that low cholesterol was associated with lower birthweight in women with BMI <25kg/m^2^, but not in women with BMI ≧25 kg/m^2^ (27). The inconsistency with our study might be because included subjects were classified into normal-weight and overweight, but no underweight women were further divided in M.Mudd et al. (27). Krstevska et al. showed that TC levels were negatively correlated, whereas LDL-c positively correlated, with LGA in women complicating diabetes (28). Adank et al. reported remnant cholesterol in early pregnancy, which was calculated by TC minus HDL-c and LDL-c, was positively associated with risk of LGA independent of pre-pregnancy BMI, but no association between lipids in early pregnancy and SGA (29). A systematic review reported a positive correlation between maternal TG and LGA, a positive correlation between maternal HDL-c and SGA and a negative correlation between maternal TG and SGA, but no other significant correlation between other lipids and LGA/SGA were found (30). The inconsistency might be explained by the variations in study design (stratifying by pre-pregnancy BMI), or different timing of tests such as early or mid-pregnancy.

On the other hand, the GWG of underweight women was significantly higher than that in normal-weight and overweight women. A previous large multi-center study demonstrated that in comparison to GWG within the IOM recommendations, GWG above was associated with increased risks for LGA and reduced risk for SGA (31). So we did a further subgroup analysis, in which the subjects in each group were divided into above and within the IOM recommendations, to eliminate the potential effects from GWG. Our results showed that the positive association between maternal TC level and LGA incidence was observed in underweight women above the IOM recommendations only, and a negative association between maternal TC level and SGA incidence was found in underweight women disregard the GWG. In addition, these associations remained after adjusting the effects of GWG, suggesting that higher maternal TC level was related to increased risk for LGA, especially in underweight women whose GWG was above the IOM recommendations, and GWG might contribute less significantly to this association.

Our findings imply that underweight women with high and low maternal TC levels during mid-pregnancy might have a higher risk of LGA and SGA respectively, and maternal TC level might be useful in identifying high-risk for LGA or SGA. Among underweight women who disregard the GWG within or beyond the IOM recommendations, low maternal TC was associated with an increased risk of SGA. Although the effects of nutrition intakes on maternal TC levels were not studied in our studies, our findings suggested that low maternal TC might reflect undernutrition and deficiency in micronutrients essential for fetal development, and undernutrition indicated by low maternal TC level during the second trimester of gestation should be avoided to prevent SGA. However, excessive maternal cholesterol might also be associated with an increased risk of LGA, especially in underweight women whose GWG were beyond the IOM recommendations. It has been demonstrated that women with higher cholesterol intake were more likely to have LGA infants than women with lower cholesterol intake, suggesting that dietary intake of cholesterol during gestation also affects fetal growth (32). Moreover, given the similar proportion of underweight and overweight women and the high risk of metabolic disorders in “metabolically obese” women, we should pay equivalent attention to pregnant women with obesity on underweight women. Therefore, lifestyle interventions for controlling gestation weight gain should be emphasized not only in overweight women or women with obesity, but also in pre-pregnancy underweight women.

On the other hand, our findings demonstrated that maternal TG level was positively correlated with the incidence of GDM, preeclampsia and preterm birth disregard the pre-pregnancy BMI, which were in accordance with previous studies (33–35). These observations suggested that metabolic management of lipids during gestation should be implemented in all pregnant women.

### Strengths and Limitations

The main strength of this study was that we divided the included subjects by pre-pregnancy BMI based on a proper criterion, and investigated the association between lipid profiles during mid-pregnancy and adverse obstetric outcomes. Associations identified in our study were more reliable after eliminating the effects of pre-pregnancy BMI and GWG. Additionally, we focused on the associations between cholesterol level and adverse obstetric outcomes in underweight women, which have not been well investigated before. Thus, findings in our study provided evidence that clinical management of lipids was also necessary for underweight women. Besides, a large sample size with more than 6, 000 subjects was also a strength of our study.

There were some limitations in our study. Demographic information including height and weight were recalled and self-reported by included subjects, which might cause recall bias in data analysis. Also, diet before the test might affect the lipid and glucose levels, supported by the observations that the amount and composition of dietary fat were associated with lipid concentrations (36, 37). Proper diet guidance before blood tests should be given to pregnant women. On the other hand, women diagnosed as GDM might voluntarily adjust their lifestyle during gestation, which might improve the pregnancy outcomes and affect the associations between lipid profiles and adverse obstetric outcomes we measured in this study.

## Conclusion and Implications

We found that the proportion of underweight women was nearly equivalent to that of overweight women, using the WHO criteria specific for Asian populations. Maternal TC levels of underweight women were significantly higher than those of normal-weight and overweight women. Besides, a positive association between maternal TC levels and incidence of LGA and a negative association between TC levels and the incidence of SGA was found in underweight but not in normal-weight or overweight women, especially among underweight women who gained weight above the IOM recommendations. Our findings provided evidence that clinic management on lipids and weight gain during gestation should also be recommended to underweight women.

## Data Availability Statement

The raw data supporting the conclusions of this article will be made available by the authors, without undue reservation.

## Ethics Statement

The studies involving human participants were reviewed and approved by The Institutional Review Board of The First Affiliated Hospital of Sun Yat-sen University. The patients/participants provided their written informed consent to participate in this study.

## Author Contributions

ZW designed the experiments. DW and WD performed experiments, analyzed data and drafted the manuscript. CD collected the data and performed experiments. HC and WZ reviewed the manuscript. BS and ZW provided the experiments with their funding. All authors read and approved the final manuscript.

## Funding

This work was supported by the National Natural Science Foundation of China (Grant No.: 81771606), Guangdong Science and Technology Department (Grant No.: 2017A020214014), Shenzhen Science and Technology Innovation Commission (Grant No.: JCYJ20170817172241688, JCYJ20180228163459314), and Clinical Medical Project 5010 of Sun Yat-sen University, China (Grant No.: 2012004).

## Conflict of Interest

The authors declare that the research was conducted in the absence of any commercial or financial relationships that could be construed as a potential conflict of interest.

## Publisher’s Note

All claims expressed in this article are solely those of the authors and do not necessarily represent those of their affiliated organizations, or those of the publisher, the editors and the reviewers. Any product that may be evaluated in this article, or claim that may be made by its manufacturer, is not guaranteed or endorsed by the publisher.

## References

[1] Poston L Caleyachetty R Cnattingius S Corvalan C Uauy R Herring S. Preconceptional and Maternal Obesity: Epidemiology and Health Consequences. Lancet Diabetes Endocrinol (2016) 4(12):1025–36. doi: 10.1016/S2213-8587(16)30217-0 27743975

[2] Razak F Anand SS Shannon H Vuksan V Davis B Jacobs R. Defining Obesity Cut Points in a Multiethnic Population. Circulation (2007) 115(16):2111–8. doi: 10.1161/CIRCULATIONAHA.106.635011 17420343

[3] Ferrara A Kahn HS Quesenberry CP Riley C Hedderson MM. An Increase in the Incidence of Gestational Diabetes Mellitus: Northern California, 1991–2000. Obstetrics Gynecol (2004) 103(3):526–33. doi: 10.1097/01.AOG.0000113623.18286.20 14990417

[4] Wang YJCL Xiao K Horswell R Besse J Johnson J H.Ryan D. And Hu G.: Increasing Incidence of Gestational Diabetes Mellitus in Louisiana, 1997–2009. J Women’s Health (2012) 21(3):319–25. doi: 10.1089/jwh.2011.2838 22023415

[5] Feig DS Hwee J Shah BR Booth GL Bierman AS Lipscombe LL. Trends in Incidence of Diabetes in Pregnancy and Serious Perinatal Outcomes: A Large, Population-Based Study in Ontario, Canada, 1996–2010. Diabetes Care (2014) 37(6):1590–6. doi: 10.2337/dc13-2717 24705609

[6] Lei Q Niu J Lv L Duan D Wen J Lin X. Clustering of Metabolic Risk Factors and Adverse Pregnancy Outcomes: A Prospective Cohort Study. Diabetes/Metabol Res Rev (2016) 32(8):835–42. doi: 10.1002/dmrr.2803 27037671

[7] Ryckman K Spracklen C Smith C Robinson J Saftlas A. Maternal Lipid Levels During Pregnancy and Gestational Diabetes: A Systematic Review and Meta-Analysis. BJOG: Int J Obstetrics Gynaecol (2015) 122(5):643–51. doi: 10.1111/1471-0528.13261 25612005

[8] Retnakaran R Ye C Hanley AJ Connelly PW Sermer M Zinman B. Effect of Maternal Weight, Adipokines, Glucose Intolerance and Lipids on Infant Birth Weight Among Women Without Gestational Diabetes Mellitus. CMAJ (2012) 184(12):1353–60. doi: 10.1503/cmaj.111154 PMC3447046 22619341

[9] Wang C Zhu W Wei Y Su R Feng H Hadar E. The Associations Between Early Pregnancy Lipid Profiles and Pregnancy Outcomes. J Perinatol (2017) 37(2):127–33. doi: 10.1038/jp.2016.191 27787507

[10] Lim JU Lee JH Kim JS Hwang YI Kim TH Lim SY. Comparison of World Health Organization and Asia-Pacific Body Mass Index Classifications in COPD Patients. Int J Chron Obstruct Pulmon Dis (2017) 12:2465–75. doi: 10.2147/COPD.S141295 PMC5571887 28860741

[11] American College of Obstetricians and Gynecologists. Committee Opinion No. 548: Weight Gain During Pregnancy. Obstetrics Gynecol (2013) 121(1):210–2. doi: 10.1097/01.AOG.0000425668.87506.4c 23262962

[12] Obstetrics Subgroup CSoO Gynecology CMA Group of Pregnancy with Diabetes Mellitus CSoPMCMA Obstetrics Subgroup Chinese Society of O Gynecology Chinese Medical A. Group of Pregnancy With Diabetes Mellitus Chinese Society of Perinatal Medicine Chinese Medical A: [Diagnosis and Therapy Guideline of Pregnancy With Diabetes Mellitus]. Zhonghua Fu Chan Ke Za Zhi (2014) 49(8):561–9. doi: 10.3760/cma.j.issn.0529-567X.2014.08.001 25354853

[13] Hypertensive Disorders in Pregnancy Subgroup CSoO Gynecology CMA. Hypertensive Disorders in Pregnancy Subgroup Chinese Society of O, Gynecology Chinese Medical A: [Diagnosis and Treatment Guideline of Hypertensive Disorders in Pregnancy (2015)]. Zhonghua Fu Chan Ke Za Zhi (2015) 50(10):721–8. doi: 10.3760/cma.j.issn.0529-567X.2015.10.001 26675569

[14] Obstetrics Subgroup CSoO Gynecology CMA. Obstetrics Subgroup Chinese Society of O, Gynecology Chinese Medical A: [Guideline of Prevention and Treatment About Postpartum Hemorrhage (2014)]. Zhonghua Fu Chan Ke Za Zhi (2014) 49(9):641–6. doi: 10.3760/cma.j.issn.0529-567X.2014.09.001 25487447

[15] Zhu L Zhang R Zhang S Shi W Yan W Wang X. *Et Al*: [Chinese Neonatal Birth Weight Curve for Different Gestational Age]. Zhonghua Er Ke Za Zhi (2015) 53(2):97–103. doi: 10.3760/cma.j.issn.0578-1310.2015.02.007 25876683

[16] Obstetrics Subgroup CSoO Gynecology CMA. Obstetrics Subgroup Chinese Society of O, Gynecology Chinese Medical A, Obstetrics Subgroup Chinese Society of O, Gynecology Chinese Medical A: [Diagnosis and Therapy Guideline of Preterm Birth (2014)]. Zhonghua Fu Chan Ke Za Zhi (2014) 49(7):481–5. doi: 10.3760/cma.j.issn.0529-567x.2014.07.001 25327726

[17] Wang C Zhu W Wei Y Su R Feng H Lin L. The Predictive Effects of Early Pregnancy Lipid Profiles and Fasting Glucose on the Risk of Gestational Diabetes Mellitus Stratified by Body Mass Index. J Diabetes Res (2016) 2016:3013567. doi: 10.1155/2016/3013567 26981541 PMC4770134

[18] Wu Y Ming WK Wang D Chen H Li Z Wang Z. Using Appropriate Pre-Pregnancy Body Mass Index Cut Points for Obesity in the Chinese Population: A Retrospective Cohort Study. Reprod Biol Endocrinol (2018) 16(1):77. doi: 10.1186/s12958-018-0397-z 30097043 PMC6087005

[19] Kulkarni SR Kumaran K Rao SR Chougule SD Deokar TM Bhalerao AJ. Maternal Lipids are as Important as Glucose for Fetal Growth: Findings From the Pune Maternal Nutrition Study. Diabetes Care (2013) 36(9):2706–13. doi: 10.2337/dc12-2445 PMC3747887 23757425

[20] Lassance L Haghiac M Minium J Catalano P Hauguel-de Mouzon S. Obesity-Induced Down-Regulation of the Mitochondrial Translocator Protein (TSPO) Impairs Placental Steroid Production. J Clin Endocrinol Metab (2015) 100(1):E11–8. doi: 10.1210/jc.2014-2792 PMC4283024 25322273

[21] Tint GS Irons M Elias ER Batta AK Frieden R Chen TS. Defective Cholesterol Biosynthesis Associated With the Smith-Lemli-Opitz Syndrome. N Engl J Med (1994) 330(2):107–13. doi: 10.1056/NEJM199401133300205 8259166

[22] Pratt JP Kaucher M Moyer E J.Richards A H.Williams H. Composition of the Human Placenta; Lipid Content. Am J Obstet Gynecol (1946) 52(4):665–8. doi: 10.1016/0002-9378(46)90135-4 21001406

[23] Sattar N Greer IA Galloway PJ Packard CJ Shepherd J Kelly T. Lipid and Lipoprotein Concentrations in Pregnancies Complicated by Intrauterine Growth Restriction. J Clin Endocrinol Metab (1999) 84(1):128–30. doi: 10.1210/jcem.84.1.5419 9920072

[24] Butte NF. Carbohydrate and Lipid Metabolism in Pregnancy: Normal Compared With Gestational Diabetes Mellitus. Am J Clin Nutr (2000) 71(5 Suppl):1256S–61S. doi: 10.1093/ajcn/71.5.1256s 10799399

[25] Schaefer-Graf UM Graf K Kulbacka I Kjos SL Dudenhausen J Vetter K. Maternal Lipids as Strong Determinants of Fetal Environment and Growth in Pregnancies With Gestational Diabetes Mellitus. Diabetes Care (2008) 31(9):1858–63. doi: 10.2337/dc08-0039 PMC2518359 18606978

[26] Kaneko K Ito Y Ebara T Kato S Matsuki T Tamada H. Association of Maternal Total Cholesterol With SGA or LGA Birth at Term: The Japan Environment and Children’s Study. J Clin Endocrinol Metab (2021). doi: 10.1210/clinem/dgab618 PMC8684489 34416000

[27] Mudd LM Holzman CB Evans RW. Maternal Mid-Pregnancy Lipids and Birthweight. Acta Obstet Gyn Scan (2015) 94(8):852–60. doi: 10.1111/aogs.12665 PMC4503474 25912426

[28] Krstevska B Jovanovska SM Krstevska SS Nakova VV Serafimoski V. Maternal Lipids May Predict Fetal Growth in Type 2 Diabetes Mellitus and Gestational Diabetes Mellitus Pregnancies. Pril (Makedon Akad Nauk Umet Odd Med Nauki) (2016) 37(2-3):99–105. doi: 10.1515/prilozi-2016-0022 27883318

[29] Adank MC Benschop L Kors AW Peterbroers KR Smak Gregoor AM Mulder MT. Maternal Lipid Profile in Early Pregnancy is Associated With Foetal Growth and the Risk of a Child Born Large-for-Gestational Age: A Population-Based Prospective Cohort Study : Maternal Lipid Profile in Early Pregnancy and Foetal Growth. BMC Med (2020) 18(1):276. doi: 10.1186/s12916-020-01730-7 33004027 PMC7532083

[30] Wang J Moore D Subramanian A Cheng KK Toulis KA Qiu X. Gestational Dyslipidaemia and Adverse Birthweight Outcomes: A Systematic Review and Meta-Analysis. Obes Rev (2018) 19(9):1256–68. doi: 10.1111/obr.12693 29786159

[31] Rogozińska E Zamora J Marlin N Betrán AP Astrup A Bogaerts A. Gestational Weight Gain Outside the Institute of Medicine Recommendations and Adverse Pregnancy Outcomes: Analysis Using Individual Participant Data From Randomised Trials. BMC Pregnancy Childbirth (2019) 19(1):322. doi: 10.1186/s12884-019-2472-7 31477075 PMC6719382

[32] de Castro MBT Farias DR Lepsch J Mendes RH Ferreira AA Kac G. High Cholesterol Dietary Intake During Pregnancy is Associated With Large for Gestational Age in a Sample of Low-Income Women of Rio De Janeiro, Brazil. Maternal Child Nutr (2017) 13(3):e12361. doi: 10.1111/mcn.12361 PMC6866211 27696759

[33] Kumru P Arisoy R Erdogdu E Demirci O Kavrut M Ardic C. Prediction of Gestational Diabetes Mellitus at First Trimester in Low-Risk Pregnancies. Taiwan J Obstet Gynecol (2016) 55(6):815–20. doi: 10.1016/j.tjog.2016.04.032 28040126

[34] Hou RL Zhou HH Chen XY Wang XM Shao J Zhao ZY. Effect of Maternal Lipid Profile, C-Peptide, Insulin, and HBA1c Levels During Late Pregnancy on Large-for-Gestational Age Newborns. World J Pediatr (2014) 10(2):175–81. doi: 10.1007/s12519-014-0488-7 24801236

[35] Wu Q Zhang L Huang L Lei Y Chen L Liang Z. Second-Trimester Maternal Lipid Profiles Predict Pregnancy Complications in an Age-Dependent Manner. Arch Gynecol Obstet (2019) 299(5):1253–60. doi: 10.1007/s00404-019-05094-z 30834968

[36] Hruby A Manson JE Qi L Malik VS Rimm EB Sun Q. Determinants and Consequences of Obesity. Am J Public Health (2016) 106(9):1656–62. doi: 10.2105/AJPH.2016.303326 PMC4981805 27459460

[37] Schwingshackl L Hoffmann G. Comparison of Effects of Long-Term Low-Fat *vs* High-Fat Diets on Blood Lipid Levels in Overweight or Obese Patients: A Systematic Review and Meta-Analysis. J Acad Nutr Diet (2013) 113(12):1640–61. doi: 10.1016/j.jand.2013.07.010 24139973

