# Supplementary material for: Higher Peripheral Cholesterol and a Positive Correlation With Risk for Large-For-Gestational-Age Neonates in Pre-Pregnancy Underweight Women
Source: Front Endocrinol (Lausanne). 2021 Nov 24;12:760934. doi: 10.3389/fendo.2021.760934 (PMC8663000; doi:10.3389/fendo.2021.760934)
Supplement: Supplementary file 1 [file Table_1.docx]

**Supplementary Table 1.** **Odds Ratios (ORs) for risks of pregnancy complications and adverse obstetric outcomes stratified by pre-pregnancy BMI adjusting gestational weight gain**

|  | GDM | | Preeclampsia | | Preterm birth | | PPH | | LGA | | SGA | |
| --- | --- | --- | --- | --- | --- | --- | --- | --- | --- | --- | --- | --- |
|  | aOR | 95% CI | aOR | 95% CI | aOR | 95% CI | aOR | 95% CI | aOR | 95% CI | aOR | 95% CI |
| Underweight women | | |  |  |  |  |  |  |  |  |  |  |
| TC | 1.05 | 0.89 – 1.23 | 0.96 | 0.64 – 1.43 | 1.10 | 0.87 – 1.40 | 1.04 | 0.77 – 1.39 | 1.30 | 0.84 – 2.02 | 0.79 | 0.66 – 0.94 |
| TG | 1.34 | 1.01 – 1.76 | 1.06 | 0.94 – 1.19 | 1.01 | 0.86 – 1.18 | 0.84 | 0.48 – 1.47 | 1.01 | 0.73 – 1.40 | 0.75 | 0.54 – 1.04 |
| HDL-c | 0.86 | 0.52 – 1.44 | 1.04 | 0.31 – 3.50 | 1.06 | 0.52 – 2.23 | 1.09 | 0.43 – 2.63 | 2.37 | 0.59 – 7.99 | 1.01 | 0.60 – 1.68 |
| LDL-c | 1.02 | 0.80 – 1.29 | 0.90 | 0.49 – 1.57 | 1.13 | 0.79 – 1.58 | 1.14 | 0.74 – 1.71 | 1.36 | 0.69 – 2.45 | 0.65 | 0.50 – 0.84 |
| TG/HDL-c | 1.48 | 1.07 – 2.16 | 1.54 | 0.94 – 2.45 | 1.04 | 0.57 – 1.56 | 0.82 | 0.32 – 1.49 | 1.00 | 0.25 – 1.92 | 0.73 | 0.44 – 1.11 |
| Normal-weight women | | |  |  |  |  |  |  |  |  |  |  |
| TC | 1.07 | 0.40 – 1.49 | 0.96 | 0.77 – 1.18 | 1.07 | 0.93 – 1.21 | 1.08 | 0.94 – 1.23 | 0.96 | 0.80 – 1.13 | 0.89 | 0.78 – 1.01 |
| TG | 1.25 | 1.12 – 1.39 | 1.28 | 1.07 – 1.49 | 1.24 | 1.09 – 1.41 | 0.96 | 0.80 – 1.13 | 1.38 | 1.20 – 1.60 | 0.89 | 0.75 – 1.05 |
| HDL-c | 0.92 | 0.69 – 1.22 | 0.88 | 0.46 – 1.66 | 0.62 | 0.41 – 0.94 | 1.12 | 0.74 – 1.69 | 0.45 | 0.27 – 0.75 | 0.85 | 0.57 – 1.24 |
| LDL-c | 1.02 | 0.95 – 1.08 | 0,93 | 0.67 – 1.07 | 1.01 | 0.88 – 1.07 | 1.01 | 0.87 – 1.07 | 0.99 | 0.78 – 1.07 | 0.85 | 0.70 – 1.01 |
| TG/HDL-c | 1.23 | 1.07 – 1.44 | 1.26 | 1.00 – 1.52 | 1.24 | 1.05 – 1.48 | 0.94 | 0.72 – 1.17 | 1.45 | 1.21 – 1.76 | 0.94 | 0.74 – 1.14 |
| Overweight women | | |  |  |  |  |  |  |  |  |  |  |
| TC | 0.93 | 0.81 – 1.07 | 0.97 | 0.77 – 1.19 | 0.93 | 0.74 – 1.14 | 1.03 | 0.80 – 1.29 | 0.97 | 0.76 – 1.21 | 0.98 | 0.74 – 1.25 |
| TG | 1.18 | 1.02 – 1.37 | 1.09 | 0.91 – 1.25 | 1.09 | 0.93 – 1.27 | 1.29 | 1.08 – 1.62 | 1.12 | 0.95 – 1.31 | 1.12 | 0.95 – 1.33 |
| HDL-c | 0.90 | 0.57 – 1.43 | 0.65 | 0.30 – 1.38 | 0.89 | 0.42 – 1.82 | 0.75 | 0.32 – 1.72 | 0.79 | 0.35 – 1.73 | 1.13 | 0.46 – 2.70 |
| LDL-c | 0.92 | 0.74 – 1.14 | 1.01 | 0.71 – 1.41 | 0.92 | 0.65 – 1.28 | 1.10 | 0.75 – 1.59 | 1.01 | 0.70 – 1.43 | 0.98 | 0.64 – 1.47 |
| TG/HDL-c | 1.27 | 1.03 – 1.60 | 1.17 | 0.89 – 1.48 | 1.13 | 0.87 – 1.44 | 1.48 | 1.14 – 2.05 | 1.24 | 0.95 – 1.58 | 1.19 | 0.90 – 1.55 |

OR: odds ratio; BMI: body mass index; GDM: gestational diabetes mellitus; PPH: postpartum hemorrhage; LGA: large for gestational age; SGA: small for gestational age; TC: total cholesterol; TG: triglycerides; HDL-c: high-density lipoprotein cholesterol; LDL-c: low-density lipoprotein cholesterol.

All aORs were adjusted for maternal age and gestational weight gain

**Supplementary Table 2.** **Odds Ratios (ORs) for risks of pregnancy complications and adverse obstetric outcomes stratified by pre-pregnancy BMI adjusting gestational weight gain**

|  | GDM | | Preeclampsia | | Preterm birth | | PPH | | LGA | | SGA | |
| --- | --- | --- | --- | --- | --- | --- | --- | --- | --- | --- | --- | --- |
|  | aOR | 95% CI | aOR | 95% CI | aOR | 95% CI | aOR | 95% CI | aOR | 95% CI | aOR | 95% CI |
| **Underweight** | | |  |  |  |  |  |  |  |  |  |  |
| Above IOM recommended GWG | | |  |  |  |  |  |  |  |  |  |  |
| TC | 0.94 | 0.47 – 1.81 | 1.69 | 0.66 – 4.36 | 2.03 | 0.72 – 6.42 | 2.09 | 0.84 – 5.59 | 2.49 | 1.12 – 6.11 | 0.28 | 0.05 – 0.90 |
| TG | 1.27 | 0.42 – 3.63 | 3.80 | 0.77 – 25.07 | 24.02 | 2.31 – 70.47 | 0.76 | 0.11 – 3.71 | 1.72 | 0.40 – 7.47 | 0.60 | 0.09 – 2.94 |
| HDL-c | 0.25 | 0.02 – 2.27 | 2.77 | 0.18 – 41.14 | 1.89 | 0.05 – 57.01 | 14.02 | 0.89 – 377.38 | 2.77 | 0.34 – 24.34 | 0.06 | 0.00 – 1.18 |
| LDL-c | 0.91 | 0.33 – 2.21 | 1.81 | 0.53 – 5.46 | 2.10 | 0.54 – 7.89 | 2.44 | 0.78 – 7.63 | 2.67 | 1.02 – 7.38 | 0.42 | 0.08 – 1.59 |
| TG/HDL-c | 1.94 | 0.34 – 10.05 | 2.56 | 0.26 – 21.34 | 16.94 | 7.12 – 73.42 | 0.13 | 0.00 – 2.60 | 1.09 | 0.12 – 7.23 | 2.11 | 0.23 – 15.58 |
| Under IOM recommended GWG | | | | | | | | |  |  |  |  |
| TC | 1.02 | 0.86 – 1.21 | 0.85 | 0.54 – 1.32 | 1.06 | 0.82 – 1.35 | 0.95 | 0.68 – 1.29 | 0.98 | 0.51 – 1.80 | 0.81 | 0.67 – 0.96 |
| TG | 1.26 | 1.02 – 1.68 | 1.05 | 0.80 – 1.17 | 0.98 | 0.66 – 1.11 | 0.83 | 0.44 – 1.10 | 0.99 | 0.32 – 1.19 | 0.76 | 0.54 – 1.02 |
| HDL-c | 0.78 | 0.45 – 1.34 | 0.85 | 0.20 – 3.12 | 1.01 | 0.46 – 2.17 | 0.74 | 0.26 – 1.95 | 2.75 | 0.43 – 12.43 | 1.09 | 0.64 – 1.84 |
| LDL-c | 0.99 | 0.77 – 1.27 | 0.74 | 0.37 – 1.42 | 1.07 | 0.74 – 1.52 | 1.00 | 0.63 – 1.56 | 0.85 | 0.31 – 2.08 | 0.66 | 0.50 – 0.86 |
| TG/HDL-c | 1.47 | 1.05 – 2.18 | 1.49 | 0.87 – 2.37 | 0.97 | 0.49 – 1.50 | 0.93 | 0.36 – 1.58 | 9.63 | 0.12 – 2.06 | 0.70 | 0.41 – 1.09 |
| **Normal-weight women** | | |  |  |  |  |  |  |  |  |  |  |
| Above IOM recommended GWG | | |  |  |  |  |  |  |  |  |  |  |
| TC | 1.25 | 1.00 – 1.56 | 0.98 | 0.65 – 1.47 | 0.96 | 0.69 – 1.33 | 1.15 | 0.87 – 1.52 | 0.82 | 0.64 – 1.04 | 0.97 | 0.70 – 1.35 |
| TG | 1.46 | 1.11 – 1.90 | 1.38 | 0.84 – 2.10 | 1.28 | 0.84 – 1.85 | 1.00 | 0.67 – 1.43 | 1.65 | 1.25 – 2.18 | 1.08 | 0.68 – 1.62 |
| HDL-c | 0.69 | 0.36 – 1.32 | 1.48 | 0.48 – 4.52 | 0.37 | 0.14 – 0.96 | 0.69 | 0.30 – 1.54 | 0.29 | 0.14 – 0.58 | 1.36 | 0.54 – 3.37 |
| LDL-c | 1.53 | 1.11 – 2.13 | 0.81 | 0.43 – 1.46 | 1.07 | 0.65 – 1.71 | 1.25 | 0.83 – 1.87 | 0.83 | 0.58 – 1.18 | 0.88 | 0.53 – 1.44 |
| TG/HDL-c | 1.63 | 1.08 – 2.44 | 1.33 | 0.59 – 2.58 | 1.77 | 0.99 – 2.98 | 1.17 | 0.65 – 1.96 | 2.53 | 1.68 – 3.84 | 0.97 | 0.45 – 1.86 |
| Under IOM recommended GWG | | |  |  |  |  |  |  |  |  |  |  |
| TC | 1.03 | 0.93 – 1.14 | 0.95 | 0.74 – 1.21 | 1.09 | 0.94 – 1.25 | 1.06 | 0.91 – 1.23 | 1.10 | 0.86 – 1.39 | 0.88 | 0.76 – 1.01 |
| TG | 1.17 | 1.05 – 1.32 | 1.25 | 1.03 – 1.48 | 1.22 | 1.07 – 1.41 | 0.95 | 0.77 – 1.14 | 1.29 | 1.06 – 1.52 | 0.90 | 0.74 – 1.06 |
| HDL-c | 0.93 | 0.67 – 1.28 | 0.68 | 0.31 – 1.48 | 0.69 | 0.43 – 1.11 | 1.34 | 0.82 – 2.18 | 0.68 | 0.31 – 1.46 | 0.79 | 0.52 – 1.21 |
| LDL-c | 1.01 | 0.92 – 1.07 | 0.97 | 0.67 – 1.09 | 1.01 | 0.86 – 1.07 | 1.00 | 0.82 – 1.07 | 1.01 | 0.01 – 1.09 | 0.85 | 0.69 – 1.02 |
| TG/HDL-c | 1.15 | 0.99 – 1.35 | 1.24 | 0.97 – 1.51 | 1.19 | 1.00 – 1.43 | 0.90 | 0.64 – 1.15 | 1.29 | 1.00 – 1.56 | 0.97 | 0.75 – 1.17 |
| **Overweight** | | |  |  |  |  |  |  |  |  |  |  |
| Above IOM recommended GWG | | |  |  |  |  |  |  |  |  |  |  |
| TC | 1.04 | 0.85 – 1.25 | 1.00 | 0.75 – 1.28 | 1.08 | 0.76 – 1.41 | 0.86 | 0.59 – 1.18 | 0.94 | 0.67 – 1.26 | 0.98 | 0.62 – 1.37 |
| TG | 1.31 | 1.02 – 1.68 | 1.30 | 0.94 – 1.77 | 1.77 | 1.23 – 2.53 | 1.24 | 0.83 – 1.76 | 1.37 | 0.98 – 1.89 | 0.77 | 0.38 – 1.37 |
| HDL-c | 1.33 | 0.66 – 2.69 | 0.46 | 0.16 – 1.24 | 1.17 | 0.35 – 3.65 | 0.53 | 0.16 – 1.60 | 0.64 | 0.21 – 1.90 | 1.12 | 0.26 – 4.33 |
| LDL-c | 1.04 | 0.75 – 1.43 | 1.15 | 0.73 – 1.73 | 1.33 | 0.77 – 2.19 | 0.87 | 0.50 – 1.45 | 0.93 | 0.56 – 1.49 | 1.00 | 0.49 – 1.89 |
| TG/HDL-c | 1.31 | 0.90 – 1.90 | 1.61 | 1.00 – 2.52 | 1.91 | 1.07 – 3.25 | 1.43 | 0.80 – 2.40 | 1.74 | 1.07 – 2.82 | 0.68 | 0.23 – 1.63 |
| Under IOM recommended GWG | | | | | | |  |  |  |  |  |  |
| TC | 0.87 | 0.71 – 1.06 | 0.91 | 0.63 – 1.29 | 0.84 | 0.62 – 1.11 | 1.34 | 0.90 – 1.99 | 1.06 | 0.73 – 1.53 | 0.99 | 0.69 – 1.41 |
| TG | 1.06 | 0.92 – 1.24 | 1.02 | 0.75 – 1.21 | 1.00 | 0.78 – 1.17 | 1.39 | 1.09 – 1.92 | 1.06 | 0.81 – 1.25 | 1.15 | 0.98 – 1.42 |
| HDL-c | 0.71 | 0.37 – 1.34 | 1.01 | 0.32 – 3.11 | 0.78 | 0.30 – 1.96 | 1.13 | 0.30 – 3.98 | 1.18 | 0.36 – 3.73 | 1.16 | 0.36 – 3.61 |
| LDL-c | 0.87 | 0.65 – 1.17 | 0.84 | 0.49 – 1.42 | 0.74 | 0.47 – 1.13 | 1.47 | 0.82 – 2.62 | 1.14 | 0.65 – 1.95 | 0.98 | 0.57 – 1.66 |
| TG/HDL-c | 1.13 | 0.91 – 1.47 | 1.01 | 0.60 – 1.36 | 1.02 | 0.70 – 1.32 | 1.61 | 1.15 – 2.59 | 1.05 | 0.63 – 1.41 | 1.25 | 0.94 – 1.72 |

aOR: adjusting odd ratios; BMI: body mass index; GDM: gestational diabetes mellitus; PPH: postpartum hemorrhage; LGA: large for gestational age; SGA: small for gestational age; IOM: Institute of Medicine; GWG: gestational weight gain; TC: total cholesterol; TG: triglyceride; HDL-c: high-density lipoprotein cholesterol; LDL-c: low-density lipoprotein cholesterol.

All aORs were adjusted for maternal age and GWG.
